# Nucleoside 2'-Deoxyribosyltransferase from Psychrophilic Bacterium *Bacillus psychrosaccharolyticus* — Preparation of an Immobilized Biocatalyst for the Enzymatic Synthesis of Therapeutic Nucleosides

**DOI:** 10.3390/molecules190811231

**Published:** 2014-07-31

**Authors:** Alba Fresco-Taboada, Immacolata Serra, Jesús Fernández-Lucas, Carmen Acebal, Miguel Arroyo, Marco Terreni, Isabel de la Mata

**Affiliations:** 1 Department of Biochemistry and Molecular Biology I, Faculty of Biology, Complutense University of Madrid, C/José Antonio Novais 2, 28040 Madrid, Spain; E-Mails: albaf@bbm1.ucm.es (A.F.-T.); jesus.fernandez2@uem.es (J.F.-L.); acebal@bbm1.ucm.es (C.A.); arroyo@bbm1.ucm.es (M.A.); 2 Department of Drug Sciences and Italian Biocatalysis Center, Università degli Studi di Pavia, Via Taramelli 12, 27100 Pavia, Italy; E-Mail: immacolata.serra@unipv.it

**Keywords:** nucleoside 2'-deoxyribosyltransferase, *Bacillus psychrosaccharolyticus*, enzyme immobilization, nucleoside synthesis, trifluridine

## Abstract

Nucleoside 2'-deoxyribosyltransferase (NDT) from the psychrophilic bacterium *Bacillus psychrosaccharolyticus* CECT 4074 has been cloned and produced for the first time. A preliminary characterization of the recombinant protein indicates that the enzyme is an NDT type II since it catalyzes the transfer of 2'-deoxyribose between purines and pyrimidines. The enzyme (*Bp*NDT) displays a high activity and stability in a broad range of pH and temperature. In addition, different approaches for the immobilization of *Bp*NDT onto several supports have been studied in order to prepare a suitable biocatalyst for the one-step industrial enzymatic synthesis of different therapeutic nucleosides. Best results were obtained by adsorbing the enzyme on PEI-functionalized agarose and subsequent cross-linking with aldehyde-dextran (20 kDa and 70% oxidation degree). The immobilized enzyme could be recycled for at least 30 consecutive cycles in the synthesis of 2'-deoxyadenosine from 2'-deoxyuridine and adenine at 37 °C and pH 8.0, with a 25% loss of activity. High conversion yield of trifluridine (64.4%) was achieved in 2 h when 20 mM of 2'-deoxyuridine and 10 mM 5-trifluorothymine were employed in the transglycosylation reaction catalyzed by immobilized *Bp*NDT at 37 °C and pH 7.5.

## 1. Introduction

Nucleoside analogs can be recognized as physiological molecules and be incorporated into DNA and RNA, consequently inhibiting cellular division and viral replication [1]. In this sense, some nucleoside analogs are actually used as therapeutic agents in inhibition of cancer cell growth, inhibition of viral replication or immunosuppression [1]. Non-natural nucleosides can be synthesized by conventional chemical methods which include many protection and deprotection steps [2], and the use of contaminant organic solvents. On the contrary, enzymatic synthesis of these compounds has arisen as a friendly environmental approach, reducing time-consuming steps and increasing stereo- and regioselectivity compared to chemical synthesis. Nucleoside phosphorylases (NPs), as well as nucleoside 2'-deoxyribosyltransferases (NDTs), are multimeric enzymes, which can be employed in the enzymatic synthesis of nucleosides by mediating the transfer of glycosyl residues to acceptor bases [3,4]. Nevertheless, NDTs (EC 2.4.2.6) offer many advantages in nucleoside analogs synthesis compared to NPs, since the former ones catalyze the one-step transfer of the deoxyribosyl moiety between 2'-deoxyribonucleosides independently of the type of base (both purines and pyrimidines) [5]. As a matter of fact, the strict substrate specificity of NPs narrows the number of their possible applications [6].

The main microbial source of NDTs for nucleoside enzymatic synthesis comes from different species of *Lactobacillus*, such as *L. helveticus* [7], *L. leichmannii*, *L. fermentum* [8], and *L. reuteri* [9]. In addition, extremophiles have also shown the presence of NDTs with applicability in nucleoside synthesis [10,11]. In this sense, cold-adapted microbial enzymes are described to be more productive at low temperatures than their mesophilic or thermophilic counterparts [12]. The psychrophilic microorganism *Bacillus psychrosaccharolyticus* (CECT 4074, ATCC 23296, DSM 6) has been reported to display nucleoside 2'-deoxyribosyltransferase activity [10], demonstrating that this facultative anaerobic Gram-positive bacterium could be a NDT source. In this sense, the genome of *B. psychrosaccharolyticus* has been recently published and the *ndt* gene that codifies a putative NDT has been identified in its genome [13].

It should be remarked that the use of immobilized enzymes could be one of the most important steps that should be pursued in order to move from a new enzyme towards an efficient biocatalyst. This aspect should be particularly considered in the case of multimeric enzymes that show a very poor stability in some of the conditions required for the development of efficient preparative processes.

Immobilization on glyoxyl-agarose might accomplish highly stabilized biocatalysts due to multipoint covalent attachment of the enzyme (via imino-bonds formation) to the support [14]. This strategy has also been successfully used for immobilization and stabilization of multimeric enzymes [14,15,16,17]. However, this interaction could be often responsible of a remarkable loss of activity as a consequence of a strong distortion of the 3D structure of the enzyme.

PEI-functionalized supports have been also recently employed for the immobilization of multimeric enzymes followed by cross-linking with aldehyde-dextran in order to prevent subunit dissociation that leads to enzyme deactivation and even product contamination [18,19]. In this case, the use of PEI-coated carriers ensures a strong ionic interaction with the enzyme avoiding distortion of its 3D structure and other limitations typical of covalent immobilization [20]. Similarly, the use of aldehyde macromolecules (such as properly oxidized dextrane) allows the stabilization of the enzyme-carrier interaction by cross-linking, preventing enzyme subunit dissociation.

In this work, cloning, production, purification and functional characterization of a novel NDT from psychrophilic *Bacillus psychrosaccharolyticus* (hereafter abbreviated as *Bp*NDT) in *E. coli* BL21 (DE3) are reported. In addition, different approaches for the immobilization of *Bp*NDT onto several carriers have been studied in order to prepare an improved biocatalyst for the one-step enzymatic synthesis of the therapeutic valuable trifluridine.

## 2. Results and Discussion

### 2.1. Cloning, Expression and Purification of Nucleoside 2'-Deoxyribosyltransferase from Bacillus psychrosaccharolyticus

**Figure 1:**
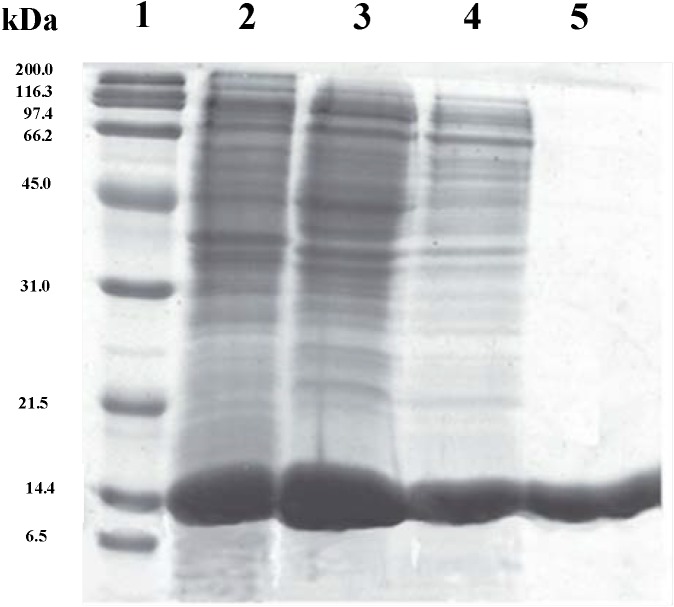
SDS-PAGE of different purification steps of *Bp*NDT produced by *E. coli* BL21 (DE3). *Lane 1*, broad range molecular weight standards from BioRad. *Lane 2*, 30 μg of protein after sonication. *Lane 3*, 30 μg of protein after anionic exchange chromatography on Bio-Scale Mini UNOsphere Q cartridge. *Lane 4*, 10 μg of protein after size-exclusion chromatography on Superose 12. *Lane 5*, 10 μg of protein after chromatofocusing on Mono P.

The *ndt* gene encoding *Bp*NDT was amplified by PCR, cloned, and overexpressed in *Escherichia coli* BL21 (DE3). The recombinant *Bp*NDT was purified from the cell extract by three chromatographic steps consisting of an anionic-exchange chromatography, a molecular size exclusion chromatography, and finally an isofocusing chromatography, as described in the Experimental Section. As result of the purification process, a total of 9.8 mg of pure *Bp*NDT per L of broth was obtained. A single protein band with an apparent molecular mass of 16 kDa was detected by SDS-PAGE (Figure 1). 

Size exclusion chromatography of pure *Bp*NDT on Sepharose 12 10/300 GL column (GE Healthcare) indicated that the native enzyme could be a homohexameric protein of 91.4 kDa, the same oligomerization state as most described NDTs [7,21,22,23,24,25], with the exception of the tetrameric *L. lactis* subsp. *lactis* NDT [26].

### 2.2. Functional Characterization of Recombinant BpNDT

Nucleoside 2'-deoxyribosyltransferases are classified into two classes depending on their substrate specificity: NDT type I (PDT) specific for purines (Pur ↔ Pur) and NDT type II (NDT), which catalyzes the transfer between purines and/or pyrimidines (Pur ↔ Pur, Pur ↔ Pyr, Pyr ↔ Pyr) [7,22,27]. In order to determine which type of 2'-deoxyribosyltransferase activity was displayed by recombinant *Bp*NDT, different reactions with ribo- and 2'-deoxyribonucleosides were performed (dUrd + Ade, Ino + Ade, Urd + Ade, dUrd + Thy). As result of experiments, *Bp*NDT should be classified as a NDT type II (NDT) since it could catalyze the transfer of 2'-deoxyribonucleosides between both purines and pyrimidines, however, it was not able to interchange the deoxyribosyl moiety between ribonucleosides (Table 1) and is free of nucleoside phosphorylases traces.

**Table 1:** Nucleoside 2'-deoxyribosiltransferase activity of *Bp*NDT.

dUrd + Ade	Ino + Ade	Urd + Ade	dUrd + Thy
+	−	−	+

Experimental conditions: 0.40 μg of enzyme were incubated at 40 °C for 5 min with 40 μL of 10 mM substrates in 50 mM MES buffer, pH 6.5.

**Figure 2:**
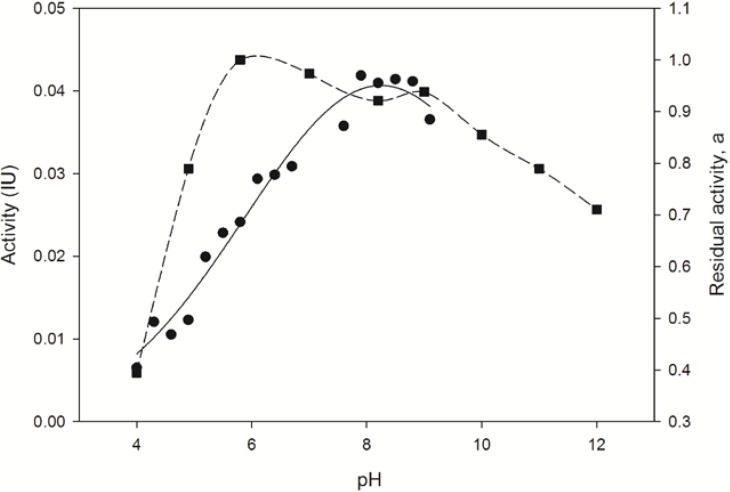
pH dependence of *Bp*NDT activity. Effect of pH on the *Bp*NDT activity (●) and stability (■) 0.4 µg of enzyme were incubated with 40 µL of 10 mM dUrd and Thy in 10 mM citrate-phosphate buffer for 5 min, 40 °C. The ionic strength (I) at each pH was adjusted to 150 mM by addition of NaCl.

The determination of the optimal conditions for activity of pure *Bp*NDT included studies of the influence of pH, ionic strength (*I*) and temperature on enzyme activity. In order to study the influence of pH on enzyme activity, the synthesis of thymidine (Thd) from 2'-deoxyuridine (dUrd) and thymine (Thy) was selected as the standard reaction due to pK_a_ values of thymine (9.7) and uracil (9.2), which guarantees the correct protonated form of nucleosides and bases in solution at the pH range assayed (from 4.0 to 9.0). As shown in Figure 2, the enzyme was stable in a pH range from 6.0 to 9.0, it retained more than 85% activity at pH 10 and it showed optimal activity at pH 8.0.

**Figure 3:**
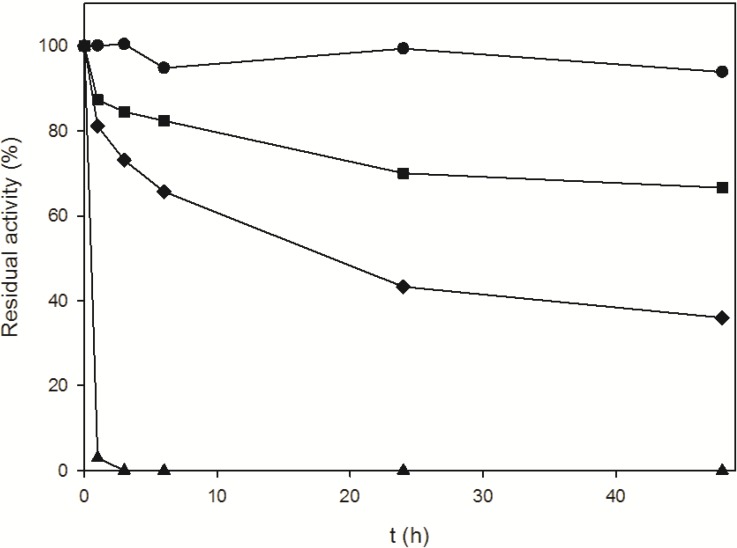
Stability in presence of 20% DMF of free *Bp*NDT (●), immobilized *Bp*NDT on PEI-agarose (▲), immobilized *Bp*NDT on PEI-agarose followed by cross-linking (1 h) with 50% oxidized aldehyde-dextran (◆), and immobilized *Bp*NDT on PEI-agarose followed by cross-linking (1 h) with 70% oxidized aldehyde-dextran (■).

Ionic strength (*I*) effect on *Bp*NDT activity was studied by adding different concentrations of NaCl in the reaction medium. Highest activity was maintained up to 1.0 M NaCl, whereas activity decreased 25% in presence of 1.5 M NaCl. As far as temperature is concerned, the *Bp*NDT activity was studied in the range from 4 to 80 °C, being most active at 50 °C (Figure 3). Thermal stability was analyzed by incubating the enzyme at temperatures ranging from 4 to 90 °C during 15 min, and the results indicated that recombinant *Bp*NDT was stable only up to 50 °C. These results are surprising for an enzyme from a psychrophilic microorganism. This behavior has also been reported for other enzymes from *B. psychrosaccharolyticus* [28], pointing out that this microorganism should be considered a psychrotolerant bacterium.

### 2.3. Immobilization of Recombinant BpNDT

Different immobilization strategies have been attempted to prepare a robust biocatalyst of the multimeric NDT from *B. psychrosaccharolyticus*, including (i) covalent attachment to glyoxyl-agarose, and (ii) ionic adsorption to supports functionalized with polyethyleneimine (PEI) and post-immobilization techniques. 

#### 2.3.1. Covalent Immobilization of *Bp*NDT on Glyoxyl-Agarose

Covalent attachment of *Bp*NDT to glyoxyl-agarose was carried out at two different temperatures (25 °C or 4 °C) and pH 10. Likewise, 2'-deoxyuridine (dUrd) was optionally incorporated as a protective additive during the immobilization process in order to avoid possible distortions of the enzyme active site structure upon interaction with the activated carrier (see Experimental Section). In alkaline conditions (pH 10), immobilization takes place between aldehyde groups of the support and ε-amino groups of lysines, mostly located on the enzyme surface. The formed imino bonds are weak Schiff bases, which are finally reduced into stable single C-N bonds by the addition of NaBH_4_.

As shown in Table 2, *Bp*NDT could be bound to glyoxyl-agarose in all cases, achieving best results when immobilization was conducted at 25 °C in absence of dUrd as additive (55.9% of immobilization yield). However, most of the immobilized biocatalysts were void of activity, with the exception of the derivative that was obtained at 4 °C, but its activity was negligible after NaBH_4_ reduction (2.6% recovered activity). High immobilization yields, but analogous enzyme inactivation, were also obtained after binding of uridine phosphorylase (EC 2.4.2.3) from *Bacillus subtilis* (*Bs*PyNP), (0% recovery) [15] and thymidine phosphorylase (EC 2.4.2.4) from *E. coli* (*Ec*TP) (6% recovery) [29] on the same support. Such drastic enzymatic loss may be ascribed to the distortion of the protein structure, or even subunit dissociation of these enzymes, caused by their multipoint attachment to highly activated glyoxyl-agarose. In the case of tetrameric purine nucleoside phosphorylase (EC 2.4.2.1) from *Bacillus subtilis* (*Bs*PNP), inosine was added in order to stabilize this multimeric enzyme during covalent attachment to glyoxyl-agarose, but enzyme binding was not achieved [15].

**Table 2:** Immobilization of *Bp*NDT on glyoxyl (covalent) and PEI (ionic adsorption) activated carriers.

Carrier	Immobilization Conditions	Immobilized Enzyme *^a^* (%)	Offered Activity (IU) *^b^*	Final Activity *^b^*	Recovered Activity (%) *^c^*
Glyoxyl-agarose	25 °C	55.9	8.6	0	0
Glyoxyl-agarose	4 °C	44.7	8.6	0.22	2.6
Glyoxyl-agarose	25 °C + dUrd *^d^*	33.8	8.6	0	0
Glyoxyl-agarose	4 °C + dUrd *^d^*	27.0	8.6	0	0
PEI-agarose	25 °C	100	11.3	11.3	100

PEI: polyethylenimine (MM 600 Da); Covalent immobilization on glyoxyl-agarose was performed at pH 10, while adsorption on PEI-carriers was performed at pH 7.5; *^a^* Relative amount of bound enzyme to the support with respect to the initial amount of enzyme prior to the immobilization process; *^b^* IU/g wet carrier; *^c^* (Activity of immobilized NDT/loaded activity) × 100; (Activity assay: synthesis of 2'-deoxyadenosine from 2'-deoxyuridine and adenine; see Experimental Section); *^d^* Addition of 2'-deoxyuridine as a protective additive during immobilization.

#### 2.3.2. Immobilization of *Bp*NDT on PEI-functionalized Supports

A different immobilization approach was chosen in order to keep the *Bp*NDT quaternary structure unaltered, in attempt to maintain the correct assembly of the oligomeric form, and thus preserving the enzyme activity as much as possible. In this sense, ion exchange of enzymes on PEI-coated supports was chosen as a simple non-distorting immobilization method that allows the full coverage of multimeric enzymes by the flexible branches of polyethylenimine. In fact, PEI is a polycationic polymer whose high density of ionized amino groups allows a strong ionic exchange with anionic groups (carboxylic groups of both aspartate and glutamate residues) of enzyme surface located in different subunits [30]. In our case, hydrophilic agarose functionalized with branched PEI (MM 600 Da) were used for *Bp*NDT immobilization at 25 °C and pH 7.5, taking into account that pI of the enzyme is 4.6. As shown in Table 2, a complete enzyme adsorption was quickly achieved (after 1 h), and enzyme activity was barely affected by the immobilization process. Our results indicate that immobilization of *Bp*NDT on PEI-coated agarose was efficiently performed without negatively affecting the oligomeric structure of the enzyme and, therefore, preserving its catalytic activity. Similar immobilization yields were obtained with trimeric *Bs*UP [15], dimeric *Ec*TP [29], and hexameric purine nucleoside phosphorylase from *Aeromonas hydrophila* (*Ah*PNPII) [17] immobilized on PEI-coated carriers, activity recovery of all these biocatalysts was higher than 85%. 

Nevertheless, immobilized biocatalysts obtained by ionic exchange of enzymes to charged supports could be characterized by a non-optimal stability and may frequently suffer from protein leakage from the carrier, causing loss of activity of the enzyme derivative and product contamination. The last fact is actually an important drawback related to the industrial application of immobilized enzymes in the production of nucleoside analogs [31,32]. In this sense, post-immobilization techniques, such as aldehyde-dextran cross-linking of adsorbed enzymes to charged supports, have been addressed as an efficient approach to overcome such problems [33]. In our case, immobilized *Bp*NDT on PEI-coated supports was further cross-linked with aldehyde-dextran in order to prevent enzyme desorption. This approach has been successfully used in the case of *Bs*PyNP [15] and *Ec*TP [29], allowing the preparation of stable biocatalysts active in enzymatic transglycosylations [34]. Aldehyde-dextran is a polyaldehyde macromolecule, which can react with both free amino groups from the enzyme and the PEI-coated support, affording a covalent multipoint cross-linking between the protein subunits and the carrier. Again, the formed covalent imino bonds are weak Schiff bases, which can be reduced with NaBH_4_ to yield single bonds [33,34]. As shown in Table 3, the influence of the oxidation degree (20%, 50% or 70%) of aldehyde-dextran (20 kDa) on the activity of the immobilized *Bp*NDT has been evaluated. Likewise, the cross-linking time of the post-immobilization process were also studied. Firstly, the overall post-immobilization process generally decreased the activity of all immobilized biocatalysts, compared to the enzyme solely adsorbed to PEI-coated supports. This effect was higher when the cross-linking time and the oxidation degree of the dextran were increased. In such conditions, the activity of these immobilized biocatalysts was low (≤0.5 IU and less than 6% recovery), as a consequence of the high number of multiple bonds between the oxidized dextran and the immobilized enzyme. In fact, a higher degree of oxidation is correlated with a higher density of aldehyde groups, which are more likely to react at a longer cross-linking time, and the result would presumably be a distorting effect on enzyme conformation. As expected, activity of immobilized enzyme on PEI-agarose was increased at a shorter cross-linking time (1 h) employing aldehyde-dextran with the lowest oxidation degree (20%).

**Table 3:** Immobilization of *Bp*NDT on PEI-functionalized agarose and cross-linking with aldehyde-dextran (MM 20 kDa).

Entry	Offered Activity Per Gram of Support (IU)	Dextran Oxidation (%)	Cross-Linking Time with Dextran (Protective Additive)	Final Activity *^a^*	Recovered Actsivity (%) *^b^*
1	6.1	20	1 h	2.3	37.7
2	6.1	50	1 h	2.2	36.1
3	6.1	50	2 h	2.0	32.8
4	6.1	50	4 h	0.3	4.9
5	8.7	70	1 h	1.8	20.7
6	8.7	70	2 h	0.5	5.7
7	8.7	70	4 h	0.3	3.4

*^a^* IU/g wet carrier; activity assay: synthesis of 2'-deoxyadenosine from 2'-deoxyuridine and adenine (see Experimental Section); *^b^* (Activity of immobilized NDT/loaded activity) × 100.

#### 2.3.3. Stability of Immobilized *Bp*NDT

The optimization of an enzyme-catalyzed synthesis of nucleoside analogs usually includes the evaluation of enzyme stability in non-physiological condition. In particular, the presence of water-miscible organic co-solvents has been evaluated, since solubility of halogenated bases and their 2'-deoxyribonucleosides counterparts can be improved in such conditions [35]. Therefore, stability of free and immobilized *Bp*NDT was studied in the presence of dimethylformamide (DMF), which is one of the most commonly used solvents for nucleoside synthesis. As depicted in Figure 3, residual activity of the free enzyme was maintained for at least 48 h in presence of 20% DMF, whereas the derivative obtained by adsorption on PEI coated carrier was completely unstable, losing almost all the activity after only 2 h. This detrimental effect, which may be related to a change in enzyme microenvironment exerted by the PEI, is progressively attenuated by increasing the oxidation degree of the aldehyde-dextran used in the post immobilization cross-linking process. In particular, the best stability was obtained using dextrane oxidized at 70% as probable consequence of a stabilization of the 3D structure of the enzyme. This immobilized biocatalyst, hereafter abbreviated as PA9*Bp*NDT, was selected for further characterization and application in the synthesis of trifluridine.

#### 2.3.4. Effect of Temperature on the Activity of Immobilized *Bp*NDT

**Figure 4:**
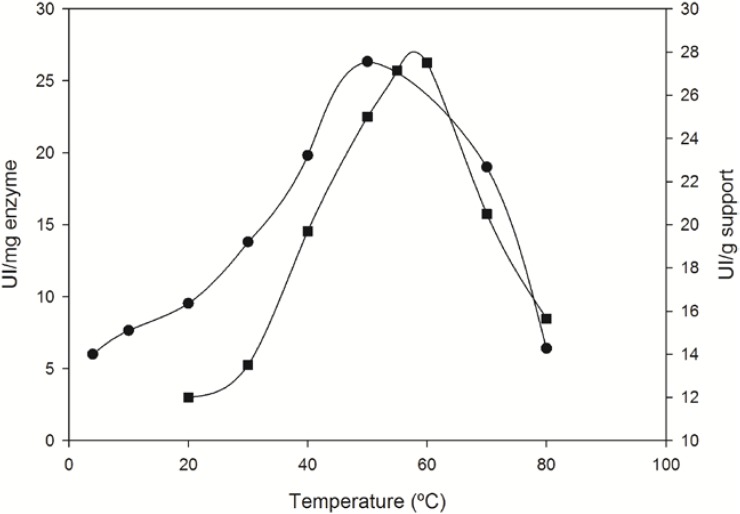
Effect of temperature on enzyme activity of free (●) and immobilized (■) *Bp*NDT (PA9*Bp*NDT biocatalyst). Enzyme activity was assayed in 50 mM HEPES buffer pH 8, as described in the Experimental Section.

Enzymatic activity of the best immobilized *Bp*NDT biocatalyst (PA9*Bp*NDT) was increased from 20 to 60 °C at pH 8.0, and then decreased very quickly as a result of the protein denaturation (Figure 4). Optimal temperature for the free soluble enzyme in the same conditions is 10 °C lower than its immobilized counterpart. This result could be another consequence of an enzyme rigidification induced by the covalent crosslinking performed with oxidized-dextran. Comparing with other immobilized NDTs, highest activity of NDT from *L. reuteri* (*Lr*NDT) covalently immobilized on epoxy-activated Sepabeads was displayed at 40 °C and pH 6.5 [36], whereas the same enzyme covalently attached to magnetic chitosan beads showed best performance at 60 °C and pH 6.5 [37]. In addition, these results indicate that soluble *Bp*NDT preserves 45% of its maximal activity at 20 °C whereas mesophilic *Lr*NDT only maintains 20% at the same temperature [9], confirming that *Bp*NDT is a more suitable enzyme for synthesizing nucleosides at low temperatures than that from a mesophilic microorganism. The experimental results of the effect of temperature were also used to estimate the activation energies (see Experimental Section). In this sense, although optimal temperature for the immobilized enzyme was 10 °C higher than that of free *Bp*NDT, the energy of activation of the immobilized *Bp*NDT (E_a_ = 23.2 kJ/mol) and free enzyme (E_a_ = 23.6 kJ/mol) were almost identical. This result indicates that the immobilization process does not affect enzyme catalysis.

#### 2.3.5. Recycling of Immobilized *Bp*NDT

Unlike free enzymes, recycling of their immobilized counterparts offers technical and economical advantages in industrial bioreactors. Therefore, the best immobilized *Bp*NDT biocatalyst (PA9*Bp*NDT) was evaluated by measuring its activity in consecutive standard assay cycles. Our immobilized biocatalyst could be recycled for at least 30 consecutive cycles in the synthesis of 2'-deoxyadenosine from 2'-deoxyuridine and adenine at 37 °C and pH 8.0, with only 25% loss of activity (results not shown).

### 2.4. Synthesis of Trifluridine Catalyzed by Immobilized BpNDT

5-Trifluorothymidine (5-tFThd, trifluridine) is a nucleoside analog primarily used in ophthalmic solutions for topical treatment of epithelial keratitis caused by herpes simplex virus (HSV) [38]. This compound also exhibits potent antitumor activity since it induces double-stranded DNA breaks and inhibits thymidylate synthase [39]. As a matter of fact, administration of the oral agent TAS-102 consisting of trifluridine in combination with a potent inhibitor of thymidine phosphorylase (which is the enzyme that degrades trifluridine), is currently undergoing a Phase III clinical trial with patients with refractory metastatic colorectal cancer (RECOURSE; NCT01607957) [40]. 

**Figure 5:**
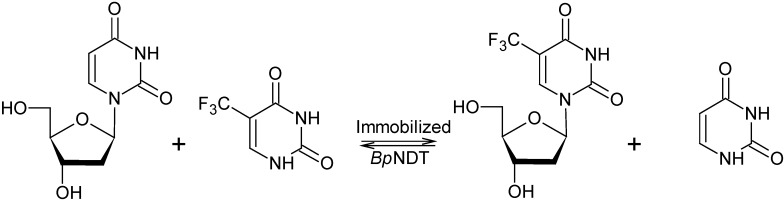
Synthesis of 5-trifluorothymidine (trifluridine) from 2'-deoxyuridine and 5-trifluorothymine by immobilized nucleoside 2'-deoxyribosyltransferase from *Bacillus psychrosaccharolyticus* (*Bp*NDT).

As depicted in Figure 5, synthesis of trifluridine from 2'-deoxyuridine (dUrd) and trifluorothymine (5-tFThy) was carried out by using the best immobilized *Bp*NDT biocatalyst (PA9*Bp*NDT) in 10 mM potassium phosphate buffer pH 7.5 at 37 °C. We analyzed the effect of substrate molar ratio, as well as substrate concentration, on conversion at 2 h reaction time and productivity (Table 4).

As observed, best conversion to trifluridine (64.4%) was achieved when 20 mM of dUrd and 10 mM 5-tFThy (2:1 ratio) were employed in the transglycosylation reaction. Although conversion was slightly decreased, productivity was higher (5.56 mM/h) when molar ratio was 1:1 at 20 mM substrate concentration compared to 2:1 molar ratio (2.92 mM/h). Similar conversions have been recently reported for the enzymatic synthesis of trifluridine catalyzed by immobilized pyrimidine nucleoside phosphorylase from *Bacillus subtilis* (57%) or thymidine phosphorylase from *Escherichia coli* (51%) requiring considerably longer reaction times [35], or by immobilized NDT from *L. reuteri* (*Lr*NDT) on magnetic chitosan beads (40%) at the same reaction time [37]. It is worth mentioning that immobilized *Bp*NDT showed a specific activity of 11.3 IU/g in trifluridine synthesis at 37 °C and pH 7.5 when molar ratio was 1:1 at 1 mM concentration of both sugar donor (dUrd) and acceptor (5tFThy). Such specific activity is approximately 140-fold higher than the one displayed by immobilized *Lr*NDT on Sepabeads in the same conditions but at 40 °C and pH 6.5 [36]. 

**Table 4:** Effect of substrate molar ratio and concentration on the enzymatic synthesis of trifluridine (5-tFThd) catalyzed by immobilized *Bp*NDT.

dUrd (mM)	5-tFThy (mM)	Conversion (%)	5-tFThd (mM)	Productivity (mM/h)
2	2	49.2	0.98	0.49
20	10	64.4	6.44	3.22
10	10	58.3	5.83	2.92
20	20	55.6	11.12	5.56

Experimental conditions: reactions were carried out by incubating the immobilized biocatalyst with 5 mL of dUrd and 5-tFThy at different concentrations in 10 mM potassium phosphate buffer pH 7.5 at 37 °C for 2 h.

## 3. Experimental Section

### 3.1. Chemicals

2'-Deoxyuridine (dUrd) was a gift from Pro.Bio.Sint (Varese, Italy) whereas adenine (Ade) and 2'-deoxycytidine (dCyd) were purchased from Sigma. 5-trifluorothymine (5-tFThy) and 5-trifluorothymidine (5-tFdThd) was from Carbosynth Ltd. (Berkshire, UK). Cross-linked 6% agarose beads (Sepharose 6BCL) were from Amersham Biosciences AB (Uppsala, Sweden). Epoxy-activated Sepabeads^®^ (EC-EP) was kindly donated by Resindion s.r.l. (Binasco, Milano, Italy). Branched polyethylenimine (PEI) with 600 Da molecular mass, and dextran with 20 kDa molecular mass was from Sigma-Aldrich (Milano, Italy). All other reagents and solvents (HPLC grade) were purchased from Sigma-Aldrich (Milano, Italy).

### 3.2. Cloning and Expression of the ndt Gene Encoding Nucleoside 2'-Deoxyribosyltransferase from *Bacillus psychrosaccharolyticus* CECT 4074

The *ndt* gene encoding nucleoside 2'-deoxyribosyltransferase from *B. psychrosaccharolyticus* was amplified by PCR using chromosomal DNA from *B. psychrosaccharolyticus* CECT 4074 as a template, obtained as described by Kieser [41]. Two synthetic primers were designed according to the sequence of *B. psychrosaccharolyticus* genome [13], 5'-CCCATGGCGAAAATTTACCTAGCTTCA CCAT-3' and 5'-GGAATTCTTATTTAACTGCTTTTAAGTACGGTTTAATAGG-3', containing the restriction sites of *Nco*I and *Eco*RI (underlined). DNA amplification was performed under standard conditions in a Mastercycler thermocycler (Eppendorf) using *Pfu* DNA polymerase. The amplified 0.43-kb product was inserted into a pET28a(+) vector, purified with the High Pure Plasmid Isolation Kit (Roche) and sequenced to confirm the absence of mutations by SECUGEN S.L. (Spain) according to dideoxy chain termination method [42] with an automated sequencer, 3730 DNA Analyzer (Applied Biosystems). This recombinant plasmid was used to transform competent *E. coli* BL21 (DE3) cells. 

### 3.3. Overproduction and Purification of Recombinant BpNDT

*E. coli* BL21 (DE3) cells harboring pET28*Bpndt* were grown on LB medium at 37 °C with kanamycin 50 μg/mL. When the culture reached 0.6 OD_600_ nm, it was induced with 0.5 mM IPTG for 2.5 h. After that, cells were harvested by centrifugation at 3500× *g* for 10 min, resuspended in 10 mM potassium phosphate buffer, pH 7.0 (buffer A) and disrupted by sonication at 4 °C by a Branson digital sonifier. The cell extract was applied onto a 5 mL Bio-Scale Mini UNOsphere Q Cartridge (BioRad) equilibrated with buffer A at a flow rate of 0.5 mL/min using a BioLogic LP chromatographic system (Bio-Rad). The retained proteins were eluted with a linear gradient from 0 to 1.0 M NaCl in the same buffer. The protein fractions containing *Bp*NDT were pooled and concentrated with polyethylene glycol 35,000 (Sigma) and loaded onto a 24 mL Superose 12 10/300 GL column (GE Healthcare) equilibrated in 50 mM potassium phosphate, pH 7.0 (buffer B) at a flow rate of 0.5 mL/min using a FPLC system (LKB-Pharmacia). The *Bp*NDT enzyme fractions were pooled and dialyzed against 0.025 M imidazol, pH 7.4 (buffer C) and loaded onto a 4 mL Mono P 5/200 GL column (GE Healthcare) equilibrated with buffer C at a flow rate of 1 mL/min. Proteins were eluted with polybuffer 74, pH 4.0 (buffer D) using a BioLogic LP chromatographic system (Bio-Rad). The protein fractions containing *Bp*NDT were detected by SDS-PAGE analysis. Electrophoresis was carried out on 15% polyacrylamide slab gel with 25 mM Tris-HCl buffer, pH 8.6, 0.1% SDS [43]. Protein concentration was determined by the Bradford method [44]. 

### 3.4. N-Deoxyribosyltransferase Assay for Soluble BpNDT

The standard activity assay was performed by incubating 5 μL of cell extract or 0.40 μg of pure enzyme with 10 mM deoxyuridine and 10 mM adenine in 50 mM HEPES buffer, pH 8.0 in a final volume of 40 μL. The reaction mixture was incubating at 40 °C, 30 r.p.m., for 5 min. Then, activity was stopped by addition of 40 μL of cold methanol in ice-bath and heating for 5 min at 95 °C as described for others NDTs [9]. After centrifugation at 9000 *×g* for 2 min, samples were half-diluted with water and the nucleoside production was measured at 254 nm by HPLC (Agilent 1100 series) with an ACE 5 μm C18-PFP column 250 mm × 46 mm (Advanced Chromatography Technologies) equilibrated with 100% trimethyl ammonium acetate at a flow rate of 1 mL/min. Elution was carried out by a discontinuous gradient: 0–10 min, 100% to 90% trimethyl ammonium acetate and 0% to 10% acetonitrile, and 10–20 min, 90% to 100% trimethyl ammonium acetate and 10% to 0% acetonitrile. Enzymatic synthesis of natural nucleosides was carried out at standard conditions described above using 10 mM of different 2'-deoxyribonucleosides and bases. Retention times for the reference natural compounds were as follows: uracil (Ura): 5.41 min; 2'-deoxyuridine (dUrd): 9.16 min; adenine (Ade): 10.14 min; 2'-deoxyadenosine (dAdo): 15.50 min; hypoxanthine (Hyp): 7.34 min; 2'-deoxyinosine (dIno): 10.95 min; thymine (Thy): 9.13 min; thymidine (Thd): 13.25; uric acid (UAc): 3.50 min. All determinations were carried out by triplicate and the maximum error was below 5%. In such conditions, one international activity unit (IU) was defined as the amount of enzyme producing 1 μmol/min of 2'-deoxyadenosine under the assay conditions.

### 3.5. Effect of Temperature on Soluble and Immobilized Recombinant BpNDT

The effect of temperature on the activity of free and immobilized recombinant *Bp*NDT was studied at different temperatures ranging from 20 to 80 °C in 50 mM HEPES buffer pH 8. The enzymatic activity was determined using the standard assay conditions described above but using the appropriate temperature. The experimental results of the effect of temperature on enzyme activity were used to estimate the activation energy by means of the equation:

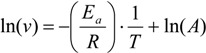
(1)
where *v* is the specific activity of immobilized enzyme, *E_a_* is the activation energy, *T* is the absolute temperature in Kelvin degrees (K), *R* is the ideal gas constant (8.314 J·K^−1^·mol^−1^) and *A* is the exponential factor. Activation energies were obtained from the section of negative slope of the plot of ln(*v*) *versus* 1/T. 

Thermal stability of free enzyme was determined by incubating pure *Bp*NDT (0.08 mg/mL) at different temperatures (from 10 to 90 °C) for 15 min. 5 μL of the enzyme were incubated on ice and the synthesis of 2'-deoxyadenosine was measured at 40 °C as described above. 

### 3.6. Effect of pH and Ionic Strength on Recombinant BpNDT Activity

Optimal pH of free enzyme was determined by measuring the activity of 0.4 μg of *Bp*NDT in 10 mM potassium citrate-phosphate buffer at different pH values (from 4.0 to 9.0) using 10 mM thymine and 10 mM 2'-deoxyuridine for the synthesis of thymidine under standard conditions. The stability at different pH values was determined by incubating pure enzyme at different pH values ranging from 4.0 to 12.0 for 15 min at 4 °C in 10 mM potassium citrate-phosphate buffer at a constant ionic strength (*I*) of 150 mM. After that, enzyme samples were adjusted to pH 8 by addition of 50 mM HEPES buffer pH 8 and *Bp*NDT activity was measured at 40 °C, as described above. The ionic strength (*I*) at each pH was adjusted to 150 mM by addition of NaCl in amounts calculated using a Visual Basic program developed in our laboratory, which allows analysis of buffer systems with up to four tetraprotic species. In addition, effect of ionic strength on NDT activity was studied by incubating 0.40 μg of enzyme with different concentrations of NaCl (0 to 1.5 M) in 50 mM HEPES buffer, pH 8.0 at 40 °C under standard conditions described for enzymatic assay.

### 3.7. Preparation of Polyethyleneimine-Functionalized Supports and Aldehyde-Dextran

Functionalization of epoxy-activated Sepabeads with PEI was performed as previously reported [15], whereas functionalization of glyoxyl-agarose [45] with PEI was carried out as previously described [18]. On other hand, aldehyde-dextran was prepared as follows: 1.67 g of dextran (MM 20 kDa) was suspended in 50 mL of distilled water and different amounts of NaIO_4 _were added to the suspension depending on the desired percentage of oxidation [46]. The solution was stirred at 25 °C for 2 h and finally dialyzed for 20 h.

### 3.8. Covalent Immobilization of BpNDT on Glyoxyl-Agarose

Five hundred milligrams of glyoxyl-agarose was added to 7.1 mL of 100 mM potassium carbonate buffer (pH 10) solution containing 0.25 mg of *Bp*NDT (4.7 IU) and incubated at room temperature or 4 °C for 3 h under mechanical stirring. In order to protect the active site, 2'-deoxyuridine (5 mM) was optionally added during the immobilization process. Once the immobilization time was finished, Schiff base-double bonds were reduced by NaBH_4_ (7.1 mg) for 30 min. Finally, immobilized *Bp*NDT was washed twice with 50 mM potassium phosphate buffer, pH 4.5, and deionized water [45]. Immobilization yield was calculated as the relative amount of bound enzyme to the support with respect to the initial amount of enzyme prior to the immobilization process, and it was calculated as the difference between offered protein and residual protein found in the filtrate and washing solutions obtained after the immobilization process. The amount of protein in these enzyme solutions was routinely determined by the Coomassie Blue method [44].

### 3.9. BpNDT Immobilization on PEI-Coated Sepabeads and PEI-Coated Agarose

*Bp*NDT immobilization was carried out by ionic adsorption of the enzyme onto PEI-functionalized supports. 500 mg of PEI-functionalized support were added to 7.1 mL of 5 mM potassium phosphate buffer (pH 7.5) solution containing 0.25 mg of *Bp*NDT (4.7 IU), and agitated by mechanical stirring for 1 h at room temperature and stored at 4 °C prior to use. Immobilization yield was calculated as the difference between offered activity and residual activity found in the filtrate and washing solutions obtained after the immobilization process.

### 3.10. Cross-Linking of Immobilized BpNDT with Aldehyde-Dextran

An amount of 0.71 mL of aldehyde-dextran (10% *v*/*v*) was added to the immobilization suspension described above. After stirring for 1 h, pH was adjusted to 10 and NaBH_4_ (1 mg/mL suspension) was added. After 30 min, immobilized *Bp*NDT was washed twice with 50 mM potassium phosphate buffer pH 4.5 and deionized water. Finally, immobilized biocatalyst was stored at 4 °C prior to use.

### 3.11. N-Deoxyribosiltransferase Assay for Immobilized BpNDT

Enzymatic activity of immobilized enzyme was measured using 25 mg of immobilized biocatalyst which was added to a 1.0 mL solution containing 10 mM 2'-deoxyuridine and 10 mM adenine in 50 mM HEPES buffer pH 8.0. Reaction mixture was incubated at 37 °C for 20 min at orbital shaking. After this reaction time, 300 μL aliquot of the supernatant was withdrawn using a pipette filter device. Then, the supernatant was analyzed by HPLC to quantitatively measure the production of nucleosides employing a Merck-Hitachi L-7100 system equipped with a UV detector L-7400 and a column oven L-7300. Reactions were analyzed with a LiChroCART^®^ RP18 (5 μm) 250 × 4.6 mm (Merck), using as mobile phase 10 mM potassium phosphate, methanol, water (90:9:1). The flow rate was fixed at 1 mL/min, and the UV detector was set at 260 nm. Retention times for the reference natural compounds were as follows: uracil (Ura): 3.5 min; 2'-deoxyuridine (dUrd): 5.5 min; adenine (Ade): 6.5 min; 2'-deoxyadenosine (dAdo): 16.5 min. All determinations were carried out by triplicate and the maximum error was below 5%. In such conditions, one international activity unit (IU) was defined as the amount of enzyme producing 1 μmol/min of 2'-deoxyadenosine under the assay conditions. In order to compare with its soluble counterpart, the free enzyme was assayed in the same standard conditions described above for the immobilized enzyme.

### 3.12. Stability of BpNDT in Organic Cosolvent Mixtures

Three hundred mg of immobilized *Bp*NDT were incubated with 2 mL of 50 mM HEPES pH 8.0 containing 20% DMF for 48 h at 37 °C; 200 µL of suspension were withdrawn at different incubation times, and activity was measured by the standard assay. For the soluble enzyme, 160 µg of *Bp*NDT were added to 2 mL of 50 mM HEPES pH 8.0 containing 20% DMF and incubated for 48 h at 37 °C. At different incubation times, 0.40 μg of enzyme were withdrawn and activity was measured by the standard assay. 

### 3.13. Recycling of Immobilized BpNDT

Immobilized enzyme (200 mg) was evaluated for nucleoside 2'-deoxyribosyltransferase activity after repeated performance of the same biocatalyst in the synthesis of 2'-deoxyadenosine from 2'-deoxyuridine and adenine. Immobilized *Bp*NDT was added to a 8 mL solution containing 10 mM 2'-deoxyuridine and 10 mM adenine in 50 mM HEPES buffer pH 8.0. After enzymatic reaction for 5 min at 37 °C and 250 rpm shaking, reaction mixture was filtered and an aliquot of the supernatant was withdrawn and half-diluted with water to be analyzed by HPLC to quantitatively measure the reaction products as described below in the analytical methods. Then, the recovered immobilized enzyme was washed three times with freshly prepared 50 mM HEPES buffer pH 8.0, and then used for another conversion cycle.

### 3.14. Enzymatic Synthesis of Trifluridine Catalyzed by Immobilized BpNDT

Trifluorothymidine (trifluridine) synthesis was performed using different concentrations of trifluorothymine and 2'-deoxyuridine (from 1 to 20 mM), which were dissolved in 5 mL of 10 mM potassium phosphate buffer pH 7.5. The enzymatic assay was started by adding 20 mg of immobilized biocatalyst when substrate concentration was 2 mM for both donor and acceptor, whereas 100 mg (displaying 1.8 IU with the standard assay) of immobilized enzyme was used at higher substrate concentrations. Reaction was performed at 37 °C for 2 h. At different reaction times, samples were withdrawn and filtered off using a pipette filter device. Then, the supernatant was analyzed by HPLC to quantitatively measure the reaction products employing a Merck-Hitachi L-7100 system equipped with a UV detector L-7400, and a column oven L-7300. Reactions were analyzed with a LiChroCART^®^ RP18 (5μm) 250 mm × 4.6 mm (Merck), using as mobile phase 10 mM potassium phosphate, methanol, water (80:18:2). The flow rate was fixed at 1 mL/min, and the UV detector was set at 260 nm. Retention times were: uracil (Ura): 3.1 min; 2'-deoxyuridine (dUrd): 4.2 min; 5-trifluorothymine (5-tFThy): 8.0 min; 5-trifluorothymidine (5-tFThd): 13.5 min. The produced nucleoside was identified by comparison of its HPLC Rt with that of authentic samples [36].

## 4. Conclusions

Here, we report, for the first time, the cloning, expression, purification and immobilization of the nucleoside 2'-deoxyribosyltransferase *Bacillus psychrosaccharolyticus* (*Bp*NDT). Best immobilized biocatalyst was obtained by adsorbing the enzyme on PEI-functionalized agarose and subsequent cross-linking with aldehyde-dextran (20 kDa and 70% oxidation degree). This novel biocatalyst could be recycled for at least 30 consecutive cycles in the synthesis of 2'-deoxyadenosine from 2'-deoxyuridine and adenine at 37 °C and pH 8.0, with a 25% loss of activity. Furthermore, the same immobilized enzyme gave high conversion yields of trifluridine at 2 h (64.4%), starting from 2'-deoxyuridine as sugar donor, and 5-trifluorothymine as base acceptor. A further detailed study on scale up would be necessary to check the applicability of this novel immobilized biocatalyst in the industrial production of these high-valuable drugs.
